# 提取肺癌小活检标本固定液中的DNA进行*EGFR*突变检测的可行性分析

**DOI:** 10.3779/j.issn.1009-3419.2019.07.05

**Published:** 2019-07-20

**Authors:** 炫之 廖, 莹莹 顾, 桔红 姜

**Affiliations:** 510120 广州，广州医科大学附属第一医院，广州呼吸健康研究院，呼吸疾病国家重点实验室 State Key Laboratory of Respiratory Disease, the First Affiliated Hospital of Guangzhou Medical University, Guangzhou 510120, China

**Keywords:** *EGFR*突变, 肺肿瘤, 标本固定液, *EGFR* mutation, Lung neoplasms, Fixation liquid

## Abstract

**背景与目的:**

表皮生长因子受体（epidermal growth factor receptor, *EGFR*）突变是非小细胞肺癌（non-small cell lung cancer, NSCLC）患者中最常见的基因突变类型，很多国际指南推荐晚期NSCLC在治疗前先进行*EGFR*突变检测，本研究探讨提取肺癌小活检标本固定液中的DNA进行EGFR检测的可行性。

**方法:**

收集取材后的肺癌小活检标本固定液，离心沉降后保存于-80 ℃冰箱。提取DNA，采用ARMS法检测*EGFR*突变状态。对比患者石蜡包埋组织*EGFR*突变状态，分析两者之间的一致性、灵敏度及特异性。

**结果:**

经临床检测石蜡包埋组织*EGFR*突变阳性28例及20例阴性病例中，其所配对的标本固定液中的分别检测出*EGFR*突变阳性20例和阴性20例，灵敏度及特异性分别为71.4%（20/28）和100.0%（20/20）。另外，同时检测了52例*EGFR*突变状态未知的石蜡包埋组织及配对的标本固定液，两种标本*EGFR*突变阳性率分别为36.5%（19/52）及28.8%（15/52），检测标本固定液的灵敏度及特异性分别为78.9%（15/19）和100.0%（33/33）。

**结论:**

提取取材后标本固定液中的DNA可能是*EGFR*突变检测的一种可行的标本来源途径。

## Abstract (en)

**Background and objective:**

Epidermal growth factor receptor (*EGFR*) mutation is the most common gene mutation in patients with non-small cell lung cancer (NSCLC). Many international guidelines are recommended to detected the *EGFR* mutation before the treatment of advanced non-small cell lung cancer. To investigate the possibility of *EGFR* mutation testing on DNA extracted from fixation liquid of lung cancer biopsy.

**Methods:**

Fixation liquid of lung cancer biopsy was collected and stored at -80 ℃ after centrifugal. DNA was extracted and *EGFR* gene mutation was detected by ARMS. Compared with *EGFR* mutation status of paraffin-embedded tissues, the consistency, the sensitivity and specificity of *EGFR* mutation testing were analyzed.

**Results:**

Among the 28 cases of *EGFR* mutation positive and 20 cases of *EGFR* mutation negative previously tested on paraffin-embedded tissue by clinic test, 20 cases with *EGFR* mutation positive and 20 cases with negative were detected by matched fixation liquid of lung cancer biopsy, respectively. The sensitivity and specificity were 71.4% and 100%. Moreover, 52 paraffin-embedded tissues and matched fixation liquid of lung cancer biopsy with unknown *EGFR* mutation status were detected, and the *EGFR* mutation positive rate were 36.5% and 28.8% respectively. The sensitivity and specificity of fixation liquid of lung cancer biopsy were 78.9% and 100.0%.

**Conclusion:**

Extracting the DNA from fixation liquid of lung cancer biopsy may be a kind of feasible way to detect *EGFR* mutation.

肺癌是常见的恶性肿瘤之一，其发病率及死亡率居我国首位^[1]^。大多数患者就诊时已处于晚期，因此临床上以多学科综合治疗为主。其中靶向治疗由于具有高选择性、低毒性和耐受性良好的优势，成为治疗肺癌特别是非小细胞肺癌（non-small cell lung cancer, NSCLC）的重要手段之一。NSCLC的靶向药物很多，分别针对不同的靶点，有表皮生长因子受体（epidermal growth factor receptor, *EGFR*）突变、动物微管相关蛋白4与间变性淋巴瘤激酶融合基因（echinoderm microtubule associated protein like 4-anaplastic lymphoma kinase, *EML4-ALK*）、间充质上皮转化因子受体（cellular-mesenchymal to epithelial transition factor, *C-MET*）扩增、鼠类肉瘤病毒癌基因同源物B1（v-raf murine sar-coma viral oncogene homolog B1, *BRAF*）突变及*C-ros*原癌基因1-受体酪氨酸激酶（*C-ros* oncogene 1-receptor tyrosine kinase, *ROS1*）重排等，其中表皮生长因子受体酪氨酸激酶抑制剂（EGFR tyrosine kinase inhibitors, EGFR-TKIs）应用最为广泛。研究表明EGFR-TKIs反应敏感突变主要是19号外显子缺失突变、21号外显子L858R突变及20号外显子插入等，而耐药突变有20号外显子T790M、19号外显子L747S及20号外显子D761Y等^[2-6]^。临床上可用于*EGFR*突变检测标本有组织标本、细胞学标本、血液标本等，其中组织标本是基因检测的金标准，然而部分病例活检组织中的肿瘤细胞量较少，进行诊断性的免疫组化之后组织不够进行分子检测。本研究中，我们拟探讨提取取材后残留的标本固定液中的DNA进行EGFR检测的可行性。分析这一方法的敏感性及特异性。

## 1 资料与方法

### 1.1 病例资料及样本的收集

收集2018年3月-2018年8月广州医科大学附属第一医院呼吸病理中心28例经临床ARMS法检测为*EGFR*突变阳性及20例突变阴性取材后的小活检标本固定液，另外收集52例*EGFR*突变未知的肺癌患者小活检石蜡包埋组织及其配对的取材后标本固定液同时进行EGFR检测。收集患者的临床病理资料，包括：性别、年龄、吸烟史、取材方式及HE切片肿瘤细胞比例等资料。入组标准：病理资料完整；无其他恶性肿瘤；取材均符合规范。

### 1.2 细胞固定液的收集

将取材后剩下的15 mL标本固定液倒入50 mL的离心管中，为了沉淀标本固定液中可能存在的游离DNA，同时加入11 mL异丙醇及650 μL的5 mol/L NaCl后，混匀，4, 000 rpm 4 ℃离心30 min，弃上清液。为了洗涤标本残留的福尔马林，加入600 μL PBS，混匀，将其转移至1.5 mL的EP管中，再加入440 μL异丙醇，混匀，13, 000 rpm 4 ℃离心30 min，弃上清，获得的细胞沉渣及DNA沉淀保存于-80 ℃冰箱。

### 1.3 DNA提取

石蜡包埋组织切片及配对的取材后标本固定液细胞沉渣及DNA沉淀均用QIAGEN DNA提取试剂盒（DNeasy Blood and Tissue Kit）提取，具体步骤如下：每份样本中加入180 μL Buffer ATL。加入20 μL蛋白激酶K，振荡混匀，56 ℃孵育至组织完全裂解，孵育过程中间断振荡。加入200 μL Buffer AL，振荡混匀，血标本需在56 ℃下孵育10 min。加入200 μL乙醇，振荡混匀。将上述混合物用移液枪移至2 mL离心管中的DNeasy Mini滤柱中，6, 000 *g*（8, 000 rpm）离心1 min，弃去滤出液及离心管。将滤柱放入新的2 mL收集管中，加入500 μL Buffer AW1，≥6, 000 *g*离心1 min，弃去滤出液及收集管。将滤柱移至新的1.5 mL或2 mL离心管。将200 μL Buffer AE加至滤柱膜中央以洗提DNA，室温（15 ℃-25 ℃）下孵育1 min。≥6, 000 *g*离心1 min。提取的DNA经过超微量分光光度计测量DNA浓度后储存于-20 ℃冰箱中。

### 1.4 *EGFR*基因突变检测

用ARMS法检测提取DNA的*EGFR*基因突变状态，包括18号-21号外显子共29个突变热点。每次实验均设突变阴性、阳性对照及空白对照孔，反应参数依据试剂盒说明书设定。根据说明书判定标准对PCR反应扩增曲线及CT值进行分析。

### 1.5 统计学方法

数据采用SPSS 20.0软件进行分析和处理，计数资料采用卡方或*Fisher*确切概率法分析。*P* < 0.05表示有统计学差异。

## 2 结果

### 2.1 患者临床特征

总共100例患者，年龄 < 60岁占51.0%（51/100），≥60岁占49.0%（49/100），男性占56.0%（56/100），女性占44.0%（44/100），有吸烟史占40.0%（40/100），无吸烟史占60.0%（60/100），经电子支气管镜取材的占44.0%（44/100），经皮肺穿刺的占15.0%（15/100），超声内镜引导下的经支气管针吸活检（endobronchailultrasound guided-transbronchial needle aspiration, EBUS-TBNA）取材占41.0%（41/100）。HE切片肿瘤细胞比例 < 50%的占68.0%（68/100），HE切片肿瘤细胞比例≥50%的占32.0%（32/100）。见表 1。

**1:** 100例肺癌患者临床资料 Clinical characteristics of 100 patients with lung cancer

Clinical features	Number
Age (yr)	
< 60	51
≥60	49
Gender	
Male	56
Female	44
Smoking history	
Yes	40
No	60
Detecting methods	
EB	44
PNLB	15
EBUS-TBNA	41
Tumor cells proportion in HE stain of biospy	
< 50%	68
≥50%	32
EB: electron bronchoscopy; PNLB: percutaneousneedle lung biopsy; EBUS-TBNA: endobronchailultrasound guided-transbronchial needle aspiration.	

### 2.2 比较经临床ARMS法检测为*EGFR*突变阳性及阴性所配对的小活检标本固定液*EGFR*突变状态

为了验证取材后的小活检标本固定液能够用于基因检测，挑选了28例石蜡包埋组织标本经临床ARMS法检测为*EGFR*突变阳性的病例，用所配对的取材后标本固定液提取的DNA检测出20例*EGFR*突变阳性。28例石蜡包埋组织标本检测12例19外显子缺失突变、6例21号外显子L858R突变及2例18号外显子G719X突变，用所配对的固定液全部检测出来，但是另外6例20号外显子插入突变及2例19外显子缺失用所配对标本固定液未能检出。见图 1及表 2。20例石蜡包埋组织标本经临床ARMS法检测*EGFR*突变阴性病例用所配对的固定液检测全部为阴性。其灵敏度为71.4%（20/28），特异性为100.0%（20/20），两者一致率为83.3%（40/48），见表 3。

**1:**
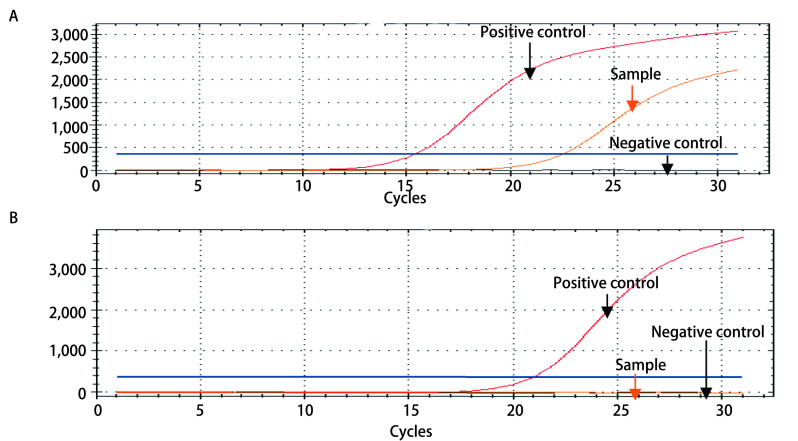
ARMS法检测*EGFR*突变代表性图：*EGFR*突变型（A）与EGFR野生型（B） Representative pictures of *EGFR* mutation detected by ARMS: *EGFR* mutation (A) and EGFR wild type (B)

**2:** 经临床ARMS法检测石蜡包埋组织为*EGFR*突变阳性配对的小活检标本固定液*EGFR*突变类型 *EGFR* mutation status of fixation liquid of lung cancer biopsy in *EGFR* mutation positive by clinical ARMS in paraffin-embedded tissue

Mutation type	Number	
	Paraffin-embedded tissue	Fixation liquid
18 exon G719X	2	2
19 exon del	14	12
20 exon insert	6	0
21 exon L858R	6	6

**3:** 比较经临床ARMS法检测石蜡包埋组织为*EGFR*突变阳性及阴性所配对的小活检标本固定液*EGFR*突变状态 Comparison of *EGFR* mutation status of fixation liquid of lung cancer biopsy in *EGFR* mutation positive and negative by clinical ARMS in paraffin-embedded tissue

Paraffin-embedded tissue	Fixation liquid		
	Mutation	Wild type	Total
Mutation	20	8	28
Wild type	0	20	20
Total	20	28	48
Sensitivity=71.4% (20 fixation liquid mutation out of 28 paraffin-embedded tissue mutation); Specificity=100.0% (all 20 paraffin-embedded tissue wild type were fixation liquid wild type); Concordance=83.3% (fixation liquid and paraffin-embedded tissue results agreed in 40 of 48 cases)			

### 2.3 比较52例肺癌小活检石蜡包埋组织及配对的标本固定液*EGFR*突变状态

为了进一步验证取材后小活检标本固定液检测*EGFR*突变的灵敏度及特异性，随机收集52例*EGFR*突变未知肺癌患者小活检石蜡包埋组织和配对的标本固定液标本，*EGFR*突变阳性率分别为36.5%（19/52）和28.8%（15/52），标本固定液DNA检测的灵敏度及特异性分别为78.9%（15/19）和100.0%（33/33），两者一致率为92.3%（48/52），见表 4。其中石蜡包埋组织共检测出19例*EGFR*突变，其中7例19外显子缺失突变、6例21号外显子L858R突变及2例20号外显子插入突变用所配对的取材后小活检标本固定液也能检测出来；3例19外显子缺失、1例21号外显子L858R突变用所配对的取材后小活检标本固定液并未检出，见表 5。

**4:** 比较52例肺癌小活检石蜡包埋组织及配对的标本固定液*EGFR*突变状态 Comparison of *EGFR* mutation status in paraffin-embedded tissue and matched fixation liquid of 52 lung cancer biopsy

Paraffin-embedded tissue	Fixation liquid		
	Mutation	Wild type	Total
Mutation	15	4	19
Wild type	0	33	33
Total	15	37	52
Sensitivity=78.9% (15 fixation liquid mutation out of 19 paraffin-embedded tissue mutation); Specificity=100.0% (all 33 paraffin-embedded tissue wld type were fixation liquid wild type); Concordance= 92.3% (fixation liquid and paraffin-embedded tissue results agreed in 48 of 52 cases).			

**5:** 52例肺癌小活检石蜡包埋组织及配对的标本固定液*EGFR*突变类型 The mutation type of *EGFR* in paraffin-embedded tissue and matched fixation liquid of 52 lung cancer biopsy

Mutation type	Number	
	Paraffin-embedded tissue	Fixation liquid
19 exon del	10	7
20 exon insert	2	2
21 exon L858R	7	6

### 2.4 100例取材后小活检标本固定液中的DNA浓度

100例取材后的小活检标本固定液标本提取的DNA后，测量DNA浓度 > 10 ng/μL有70例，5 ng/μL-10 ng/μL有18例， < 5 ng/μL有12例。石蜡包埋组织EGFR检测结果与所配对的标本固定液检测结果相一致的88例病例中，其标本固定液DNA浓度 > 10 ng/μL有68例，5 ng/μL-10 ng/μL有16例， < 5 ng/μL有4例。EGFR检测不一致的12例病例中，其对应的标本固定液DNA浓度 > 10 ng/μL有8例，5 ng/μL-10 ng/μL有2例， < 5 ng/μL有2例。*EGFR*突变检测是否一致与DNA浓度有统计学差异，DNA浓度高，*EGFR*突变检测结果一致较高，见表 6。HE切片肿瘤细胞比例与标本固定液提取的DNA浓度有统计学差异，HE切片中肿瘤细胞比例相对较高的病例，其标本固定液提取的DNA浓度越高，见表 7。

**6:** 取材后小活检标本固定液中提取的DNA浓度与*EGFR*检测结果的关系 The correlation between *EGFR* results and the concentration of DNA extracted from fixation liquid of lung cancer biopsy

DNA concentration (ng/*μ*L)	Number			*P*
	Conformity	Inconformity	Total	
< 5	4	8	12	
5-10	16	2	18	< 0.001
> 10	68	2	70	
Total	88	12	100	

**7:** 肺癌患者小活检HE切片肿瘤细胞比例与标本固定液中提取的DNA浓度的相关性 The correlation between tumor cells proportion of HE stain and the concentration of DNA extracted from fixation liquid of lung cancer biopsy

DNA concentration (ng/*μ*L)	Tumor cells proportion of HE stain			*P*
	< 50%	≥50%	Total	
< 5	22	1	23	
5-10	12	7	19	0.034
> 10	45	13	58	
Total	79	21	100	

### 2.5 100例肺癌患者小活检石蜡包埋组织及配对的标本固定液EGFR与临床特征之间的关系

100例肺癌患者小活检石蜡包埋组织及配对的标本固定液，结果两者*EGFR*突变与年龄、性别及吸烟史有统计学意义（*P* < 0.05），在年龄 < 60岁、无吸烟史、女性肺癌患者中*EGFR*突变率更高，而与取材方式及HE切片肿瘤细胞比例无统计学意义（*P* > 0.05），见表 8。

**8:** 100例肺癌患者小活检石蜡包埋组织及配对的标本固定液*EGFR*基因突变与临床特征之间的关系 The correlation between *EGFR* mutation status and clinical features of 100 paraffin-embedded tissue and matched fixation liquid of lung cancer biopsy

Clinical features	Paraffin-embedded tissue		*P*	Fixation liquid		*P*
	Mutation	Wild type		Mutation	Wild type	
Age (yr)			0.044			0.031
< 60	29	22		23	28	
≥60	18	31		12	37	
Gender			0.003			0.018
Male	19	37		14	42	
Female	28	16		21	23	
Smoking history			0.018			0.032
Yes	13	27		9	31	
No	34	26		26	34	
Detecting methods			0.184			0.178
EB	21	23		16	28	
PNLB	10	5		8	7	
EBUS-TBNA	16	25		11	30	
Tumor cells proportion in HE stain of biopsy			0.400			0.208
< 50%	30	38		21	47	
≥50%	17	15		14	18	

## 3 讨论

近年来，随着肿瘤驱动基因与临床病理关系的日趋明晰，尤其分子靶向治疗的发展，使得肺癌的治疗模式发生了很大变化。分子靶向治疗由于具有高选择性、低毒性和耐受性良好的优势，成为治疗肺癌特别是NSCLC的重要手段之一。NSCLC治疗的靶向药物很多，有EGFR抑制剂、ALK抑制剂、BRAF抑制剂、ROS1抑制剂等，分别针对不同的靶点。各个靶点在NSCLC中发生的突变频率各不相同，其中*EGFR*突变率最高，约为24.5%-36.2%^[7, 8]^，*EML4-ALK*融合基因约为3.3%-4.9%^[8, 9]^，*BRAF*突变约为3%-4% ^[7, 10]^，*ROS1*重排约为1%-2%^[11, 12]^。

靶向治疗之前需要进行基因检测。基因检测的标本有三类：细胞学标本、血液标本及组织标本。它们各自都有优缺点。细胞学标本优点是创伤较少，缺点是并不是所有患者都有胸腹水，另外细胞学标本易受标本内非肿瘤细胞的干扰，而且其获取的DNA量极少，极易出现假阴性的情况。血液标本优点是获取容易、无创，能够克服肿瘤的异质性，可以反映整个肿瘤的突变变化；缺点就是血液中的循环肿瘤DNA（circulating tumor DNA, ctDNA）含量极少，ctDNA因检测方法的不同其灵敏度差异较大，这都限制了外周血在肿瘤分子检测方面的应用^[13-15]^。组织标本所含的肿瘤细胞多，特异性及灵敏度高，是基因检测的金标准。但是，部分病例小活检取的组织较少，而且病理诊断过程中免疫组化需要切除部分组织，另外靶向治疗需要检测的靶点多，导致部分病例的组织不够进行某些靶点分子检测。为了增加病人靶向治疗之前的分子检测标本来源，我们首次提出利用取材后残留的标本固定液中的DNA进行EGFR检测，探讨其可行性。

取材后残留的标本固定液存在微小的组织、悬浮细胞及游离的DNA，其中微小组织及悬浮细胞可以通过离心获取，但是游离的DNA单靠离心是无法沉淀的，为此我们在离心之前加了异丙醇及高浓度的NaCl溶液沉降游离的DNA。100例标本中提取的DNA浓度 > 10 ng/μL有70例，5 ng/μL-10 ng/μL有18例， < 5 ng/μL有12例。

在本研究中，所有的患者均采用了ARMS法进行*EGFR*突变检测。ARMS法对肿瘤DNA含量的要求较低，甚至只需1%即可，方法简单易行，特异性及敏感性都较高，因而广泛使用^[16]^。本研究先检测了28例经临床检测为*EGFR*突变阳性病例所对应的标本固定液中的DNA，其中检测出*EGFR*突变阳性20例；然后检测20例经临床检测为*EGFR*突变阴性病例所对应的标本固定液中的DNA，结果均为阴性，其灵敏度及特异性分别为71.4%和100.0%，一致率为83.3%。为了排除事先知道*EGFR*突变状态所引起的选择性偏倚，我们后来随机选取52例*EGFR*突变未知的肺癌患者石蜡包埋组织及配对的标本固定液标本同时进行检测，结果石蜡包埋组织检出突变型19例（19/52），标本固定液中检出突变型15例（15/52），标本固定液检测的灵敏度及特异性分别为78.9%和100.0%，两者一致率为92.3%。前者灵敏度略微低于后者灵敏度，而特异性都为100.0%，表明利用取材后的小活检标本固定液进行EGFR检测是一种可行的途径。

我们在这100例肺癌患者中，总共发现12例患者配对的样本结果不一致，其中石蜡包埋组织EGFR检测为阳性突变，但是所配对的标本固定液检测结果全为阴性。分析其标本固定液中的DNA浓度，其中8例（66.7%）DNA浓度低于5 ng/μL，2例（16.7%）DNA在5 ng/μL-10 ng/μL，而在*EGFR*检测结果相一致的88例标本固定液中，68例（77.3%）DNA浓度大于10 ng/μL，只有4例（4.5%）DNA浓度小于5 ng/μL，由此可见，标本固定液中的DNA含量过少是造成组织样本检测不一致的主要原因。但是配对样本结果不一致中仍有2例（16.7%）DNA浓度高于10 ng/μL，可见除了标本固定液中的DNA含量过少是组织样本检测不一致的原因外，其中肿瘤细胞DNA含量低于ARMS法检测的阈值或许也是其原因之一^[17]^。为了探明小活检标本固定液未检出的*EGFR*突变是DNA浓度过低导致的，还是其他原因引起的，条件许可时可进行更敏感的微滴度PCR方法或二代测序方法。另外我们分析了HE切片肿瘤细胞比例与标本固定液提取的DNA浓度之间是否存在统计学差异，结果发现HE切片中肿瘤细胞比例相对较高的病例，其标本固定液提取的DNA浓度越高。

总而言之，提取肺癌小活检标本固定液中的DNA进行*EGFR*突变检测可能是一种可行的*EGFR*突变检测标本来源。我们将进一步验证此标本来源进行其他靶向治疗基因检测的可行性。
